# The Strategy of How to Deeply Integrate Technology and Finance in the Internet Environment

**DOI:** 10.1155/2022/5018160

**Published:** 2022-07-01

**Authors:** Yunyan Xu

**Affiliations:** Zhengzhou Shengda University, Zhengzhou 451191, China

## Abstract

To solve some problems existing in the mechanism of the combination of science and technology and finance, this study studies the deep integration of science and technology and finance combined with the Internet. At present, there are many problems in the development of science and technology finance in terms of bank credit, capital market, service system, and risk supervision. To solve these problems, we need to innovate bank credit products and service modes, expand the breadth and depth of capital market services, establish a new science and technology financial service system, improve science and technology financial supervision regulations and means, and comprehensively promote the deep integration of science and technology and finance in the new era. Based on this, this study first expounds on the importance of the deep integration of science and technology and finance, comprehensively analyzes the problems existing in the deep integration of science and technology and finance, and finally puts forward the construction strategy of science and technology and finance integration platform. In the development of market economy, the deep integration of science and technology and finance and the development of platform model effectively improve the innovation ability of science and technology enterprises. The research shows that the high integration of science and technology and finance is the inevitable trend of the future development of the economic market. Therefore, enterprises need to constantly improve their financial operation ability, pay attention to the ways and means of the development of science and technology finance, strengthen the construction of their own platform, improve their operation ability, and improve their development status, so that enterprises can obtain a higher position in the market competition and achieve the purpose of sustainable development.

## 1. Introduction

**Figure 1:**
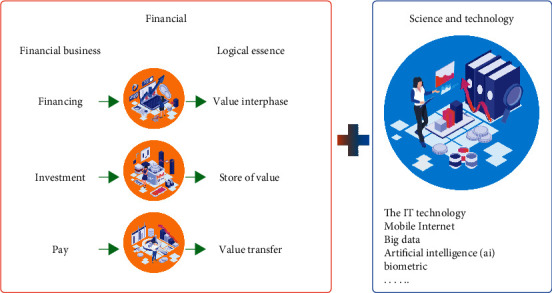
Integration of technology and finance.

Science and technology and finance are the two core elements of economic development. Scientific and technological innovation is inseparable from the support of modern finance. Modern finance expands business and improves efficiency with the help of technological innovation. The two permeate and promote each other, as shown in Figure 1. The deep integration of science and technology and finance is an important means to realize innovation-driven development. It is particularly necessary for the growth of science and technology enterprises, the transformation and upgrading of economic structure, and the construction of an innovative country. In recent years, with the rapid development of science and technology finance, the allocation of financial resources to the field of science and technology, the penetration of technological innovation into the modern financial industry, and the deepening integration of science and technology finance, there are still many problems in the actual development process, mainly in the following aspects: there are deficiencies in the bank credit model [1]. At present, the capital sources of enterprises are mainly self-raised funds and bank loans, while traditional banks still tend to give priority to provide loan financing services for large enterprises and large projects. The loan evaluation method is mainly to measure the collateral, future cash flow, and loan risk of large enterprises. For high investment and high-risk technology-based small- and medium-sized enterprises, the bank credit supply structure is single, and the innovation of product and service mode lags behind, resulting in the market that is not active enough to meet the diversified credit demand, and insufficient attention is paid to their intangible assets such as intellectual property rights, which is not conducive to the loans of high-tech enterprises. The development of the capital market system is not perfect. The capital market consists of an on-site market and an off-site market [2]. Among them, the development of the main board market is relatively mature, the capital markets at other levels are relatively backward, the GEM market is still in the primary development stage, the share transfer system of small- and medium-sized enterprises has just started, the development of property right trading market and OTC market is slow, and the development of various capital markets is uneven. Due to the high standard and threshold of main board listing, it is difficult for high-tech small- and medium-sized enterprises to go public and finance. At present, the approval system is implemented for security issuance, which focuses on the business status, profitability, and development prospects of enterprises, and does not pay attention to the R&D ability, intellectual property rights, and other intangible assets of enterprises. It is not applicable to high-tech enterprises with uncertainty and no profit [3]. The scientific and technological financial service system is not perfect. The existing science and technology intermediary services such as science and technology insurance, credit guarantee, credit evaluation and investment, and financing consulting are still in their infancy. The number and scale of service institutions are limited, there is a shortage of professionals, the ability to provide all-round services is weak, the degree of specialization is not high, the knowledge and experience in the professional field are insufficient, and there is a lack of effective guidance. The imperfect scientific and technological information sharing platform and intermediary service system make it difficult to provide professional and personalized financial services for scientific and technological enterprises, which hinders the information coupling of scientific and technological enterprises and financial institutions.

## 2. Literature Review

Jerene and Sharma said that the support of finance for the development of science and technology industry and the role of science and technology in financial reform are organically combined in social progress and economic development. With the penetration of science and technology into all aspects of economic life, scientific and technological growth will be the foundation and guarantee of future society [4]. Therefore, Jeswari and Krishnan believe that the new category of “science and technology finance” formed by the organic integration of science and technology and finance can more clearly and prominently express the mutual benefit and interaction between science and technology and finance and can more clearly reflect the characteristics of the operation mode of the new economy [5]. Yao et al. believe that finance needs effective innovation to achieve support for scientific and technological development. At the same time, the scientization of finance itself helps to further promote technological innovation and scientific and technological progress, and effective scientific and technological growth will also bring more efficient financial development [6]. Okere and Chen believe that science and technology finance is the deep integration of science and technology and finance, which is not only the focus of China's financial development in the future but also the requirement to improve the scientific and technological level of China's economic development [7]. Zdurak believes that the integration of financial innovation and scientific and technological innovation is the main embodiment of scientific and technological finance. Under the Internet environment, effectively using Internet thinking to promote the deep integration of financial innovation and scientific and technological innovation is a new choice for the rapid solution of the traditional bottleneck of China's scientific and technological finance. In the Internet environment, financial innovation is very beneficial to the deep-seated solution of the organic integration of science, technology, and finance [8]. Rahardjo et al. said that the organic integration of science, technology, and finance has been continuously strengthened and enriched in both the financial system reform and the science and technology system reform, mainly experiencing the development from single line to multilevel and from administrative system to marketization [9]. Arneret al. believe that with the support of relevant institutions such as banks, insurance, securities, and trusts, multilevel service systems such as loans, finance, capital market, venture capital, and insurance have been preliminarily established [10]. However, Velu and Narmada believe that the current credit investigation system is relatively lacking. In terms of science and technology financing, the lack of credit environment and information is still the main problem [11]. Kireyeva et al. said that at present, the high-tech zones and science parks in many cities have built relevant service platforms such as science and technology financial service groups, science and technology financial service centers, and science and technology venture capital groups [12]. Moreover, the credit service system of small- and medium-sized enterprises has also begun to be built. However, Afroz et al. said that there is still a lack of deep-seated integration, and there is still a lot of room for the function of the financial market [13].

## 3. Method

### 3.1. Demand Model of R&D Subject

Why and when will R&D subjects carry out R&D activities? Obviously, it can also be studied from the perspective of its needs. Both individual needs and organizational needs have two aspects. One is the need for survival, that is, the basic need to maintain survival (including individuals in the organization and the organization itself). This requires that the R&D subject must have a certain income or rate of return. When the income or rate of return is lower than the social average, the organization is faced with being eliminated, and the individual means a lower living standard and an unbalanced mentality. Therefore, this is an instinctive need and unconditional. The main way to meet it is to obtain more income [14]. Therefore, from the perspective of the capital scale of R&D investment, it should not only make up for the cost of R&D investment but also make the R&D subject have benefits beyond the cost. Another demand of R&D subject is spiritual demand. For organizations, obtaining honor and status is extremely beneficial to the improvement of the external environment of the organization, which will bring some hidden benefits or hidden cost reduction. For individuals, it also includes the psychological satisfaction brought by the respect of others [15].

  Assumption: *V* is the average social (or industrial) income
 
*R* is the current income of R&D individuals
 
*R* is the expected rate of return of individual R&D investment
 
*P* is the probability that the individual income of R&D decreases and is lower than the average income of society (or industry) (0 < *p* < 1)
  Let *X*=*PR*/*V*; *X*′=*PR*/*V*+*r*

Then, when *X* < 1, it means that the R&D subject has low income or certain risk, and the R&D subject has the enthusiasm to change the low income through *R*&D. The smaller the *X*, the greater the enthusiasm. On the contrary, when *X* ≥ 1, the greater the *x*, the less the enthusiasm. However, if the total income of the *R*&D subject after R&D cannot make *X*′ ≥ 1, it indicates that the total scale of R&D investment is not enough, the enthusiasm of the *R*&D subject will be greatly damaged, and the possibility of moral hazard will be amplified. *P* reflects the risk level of realizing the average social income, or the intensity of market competition [16]. Therefore, this study constructs the demand model of *R*&D subject, which can be written as follows:

(1) $$\begin{array}{c} f\left(X{}^{\prime }\right)=1/X{}^{\prime }+X{}^{\prime }\left(X{}^{\prime }>0\right). \end{array}$$

Interpretation of the model is as follows:

1. The first part of the model represents the survival needs of R&D subjects and shows that the survival needs weaken with the growth of R&D subjects' income and the weakening of competition intensity. When *X*′ approaches 0, survival demand 1/*X*′ approaches *∞*, while spiritual demand *X*′ approaches 0. It shows that due to the low income of R&D subjects, they have a strong desire to increase income. At this time, money and material stimulation are the most sensitive and effective. Honor, status, and respect cannot solve the problem of survival and have little effect on spiritual motivation.
2. The latter half of the model represents the spiritual demand and shows that the spiritual demand does not exist independently, which increases with the growth of the income of the R&D subject. When *X*′ approaches *∞*, survival needs approach 0, while spiritual needs *X*′ approach *∞*. It shows that the focus of R&D individuals turns to spiritual satisfaction such as honor, status, and social respect. In this case, they are not sensitive to money and material stimuli [17]. The incentive effect is not good; the desire to enjoy the spirit of status, honor, and social respect is strong, the stimulus response is sensitive, and the incentive effect is remarkable.
3. When *X*′=1 and 1/*X*′=*X*′, survival needs and spiritual needs are equal, and the value of demand function in this area is the smallest, indicating that the material incentive and spiritual incentive of R&D subjects in this area are effective. *X*′ < 1 focused on survival needs, and *X*′>1 focused on spiritual needs.
4. The demand function is always positive, and *f*(*X*′)=1/*X*′+*X*′ ≥ 2 indicates that the demand of the R&D subject always exists, and the incentive is always necessary and effective.

Since economic value added (EVA) was proposed in the 1980s, it has gradually been widely used since the mid-1990s. It has become an important supplement to the traditional performance measurement index system.

EVA of the enterprise = net operating profit after tax - total capital cost EVA of the enterprise R&D entity can be calculated with the following equation:

(2) $$\begin{array}{c} {\text{EVA}}_{ij}=S_{ij}-C_{ij}-K_{ij}, \end{array}$$

where *S* is the R&D revenue, *C* is the R&D cost, and *K* is the capital cost; the subscript *i* represents a certain R&D project, and *j* represents a certain year.

Since the R&D funds mainly come from self-owned funds, it is relatively simple to estimate the capital cost. The key is how to estimate R&D revenue and R&D cost.

#### 3.1.1. Estimation of R&D Revenue

From the calculation method of EVA, EVA is historical rather than forward-looking, which is suitable for post-evaluation, and the R&D income is in line with this characteristic. The value of R&D achievements is not reflected in the current period, but in the future market application [18]. Therefore, in the current period of successful R&D, EVA is not calculated, but the R&D income and EVA are calculated in the first year when the R&D results are transformed into market goods. Generally, a certain proportion of sales can be used as the R&D income of each period, as shown in the following equation:

(3) $$\begin{array}{c} S_{ij}=P_{ij}\times R_{i}, \end{array}$$

*S*_*ij*_ is the R&D income of a scientific and technological achievement in *J* years, *P*_*ij*_ is the sales income of a scientific and technological achievement product in *J* years, and *R*_*i*_ is the share of R&D income in the sales income. It can be apportioned according to the proportion of R&D cost investment in the total investment cost.

#### 3.1.2. R&D Cost Estimation

The investment of enterprises in R&D is a long-term investment. The R&D process is actually the process of transforming tangible capital such as R&D resources into intangible capital such as new technology and knowledge. Therefore, in the calculation of R&D cost, it should be considered to allocate R&D investment year by year in the future. The starting time and years of allocation should vary according to the type of R&D project. Generally speaking, the starting time should be considered when the product market is relatively stable and the quality risk is small. In addition, the sharing starting time and sharing period of product innovation with large investment scale, great difficulty, and long time are long [19].

#### 3.1.3. Consider the Time Value of Money

When calculating the EVA of the R&D entity, since the cost input and income are not at the same time, they are calculated afterwards. Therefore, if the R&D cost or capital cost is calculated, the time value of money should be considered, so compound interest calculation is required.

#### 3.1.4. EVA Bonus Incentive

The EVA of R&D subject is the contribution of R&D subject to enterprise value, which should be distributed between enterprise and R&D subject. Bonus *A* for R&D subject can be calculated according to the following formula:

(4) $$\begin{array}{c} A_{ij}={\text{EVA}}_{ij}\times L_{j}. \end{array}$$

Among them, *L*_*j*_ is the proportion of R&D entities sharing EVA in *J* years, which can decrease year by year. According to the incentive theory, the incentive effect of short-term income is more significant than that of long-term income. Therefore, this measure will help to improve the incentive effect for scientific and technological personnel and help to balance the impact of increasing sales on bonuses with product growth. In addition, when EVA_*ij*_ − EVA_*i*,*j*−1_ is negative, it means that EVA decreases and the contribution of products to enterprise value decreases. New innovation should be considered. At this time, *L*_*j*_=0, and the R&D subject will no longer enjoy EVA bonus.

There are many factors affecting whether the R&D subject makes independent R&D investment, but ultimately depends on the value of the project, which is affected by the technical span of the project [20]. The traditional net present value method (NPV) can be used to construct the R&D investment decision model. An important assumption of NPV method is that the cash flow of a project occurs within a predictable range and is then discounted to its present value. Investment decisions must be made now, either immediately or forever.

Obviously, there are two possible options:

1. Expansion or Contraction Options—when *T*_0_'s investment is completed, it is found that the project has a very good prospect in *T*_*m*_, such as strong market demand and broader and favorable utilization of achievements. Obviously, managers will choose the input of *I*(*T*_*m*_) and increase the input of Δ*I*(*T*_*m*_). On the contrary, if it is found that the prospect is not good, investors will tighten or cancel *I*(*T*_*m*_)'s investment.
2. Abandonment or Postponement of Option—if the project is found to have abandonment value *V*′ in *T*_*m*_ (e.g., selling the early results of the project has more economic value, finding more favorable alternative projects, and the project results have no market or have been developed ahead of other investors), abandoning the project is an effective choice for the manager. It is also possible to wait in phase *T*_*m*_ to receive more information for further decision-making. At this time, the input of phase *m* is *I*(*T*_*m*_)=0.

When there are expansion or contraction options, as shown in the following equations:

(5) $$\begin{array}{c} \text{NPV}=-I\left(T_{0}\right)-\frac{I\left(T_{m}\right)}{{\left(1+k_{d}\right)}^{m}}+\sum_{t=1}^{n}\frac{C_{t}}{{\left(1+k_{d}\right)}^{t}}, \end{array}$$

(6) $$\begin{array}{c} \text{MO}=P\left[-\Delta I\left(T_{m}\right)+\sum_{t=m+1}^{n}\frac{\Delta C_{t}}{{\left(1+k_{d}\right)}^{n-m}}\right]\frac{1}{{\left(1+k_{d}\right)}^{m}}. \end{array}$$

Then, project value *V*=NPV+MO.

When there is a waiver or delay option, the project value is shown in the following equation:

(7) $$\begin{array}{c} V=-I\left(T_{0}\right)+\sum_{t=1}^{m}\frac{C_{t}}{{\left(1+k_{d}\right)}^{t}}+\frac{V{}^{\prime }}{{\left(1+k_{d}\right)}^{m}}. \end{array}$$

Whenever the project value *V* ≥ 0, the project is feasible. Obviously, the above methods provide a feasible scheme for science and technology management. It can assess the sustainability of existing science and technology projects, that is, decide to continue the project or abandon or delay it, and use the transferred funds for other projects. When the abandoned value has a higher present value than the remaining future cash flow, it indicates that it is an economically infeasible project [21].

Generally, the choice of technology span is determined in the early stage of R&D investment. Therefore, only one-phase model needs to be studied. The value *V* of R&D projects can be measured by the following equation:

(8) $$\begin{array}{c} V=-I_{0}+\left[\sum_{t=1}^{T}\frac{P_{t}}{{\left(1+k_{d}\right)}^{t}}\right]\times q, \end{array}$$

where *I*_0_ is the technology investment and *P*_*t*_ is the risk return of phase *t*. *q* is the R&D risk adjustment coefficient, 0 < *q* < *I*, which is a function of the investment level of scientific and technological resources *x*_1_, the technological span *x*_2_, the R&D ability *x*_3_ of the R&D subject, and the incentive management mechanism *x*_4_. Given *x*_1_, *x*_3_,  and *x*_4_, the size of *q* depends on the technological span. The greater the technological span, the smaller the probability of R&D success and the smaller the R&D risk adjustment coefficient; the smaller the technological span, the greater the R&D risk adjustment coefficient. As shown in the following equation:

(9) $$\begin{array}{c} q=f_{1}\left(x_{2}\right). \end{array}$$

At the same time, risk return is a function of market input level *y*_1_ and technology span *x*_2_. Given *y*_1_, the greater the technical span, the greater the risk return, as shown in the following equation:

(10) $$\begin{array}{c} p=f_{2}\left(x_{2}\right). \end{array}$$

Equation (9) and equation (10) are substituted into equation (8), and through the following equation:

(11) $$\begin{array}{c} \frac{\partial \left(1/V\right)}{\partial x_{2}}=0. \end{array}$$

Then, we can find *x*_2_.

### 3.2. Internet Finance and Technology Finance under the Thinking of Investment Loan Linkage

**Figure 2:**
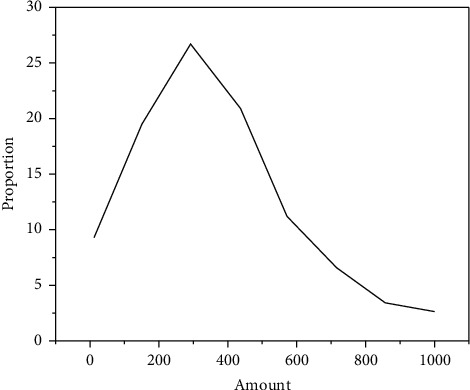
Survey results of financing needs of merchants on a platform (%, 10000 yuan).

From the essence of economics, commercial banks are typical individual enterprises, which operate independently, take responsibility for their own profits and losses, and pursue profit maximization. For individual customers or corporate customers with more assets, it can bring huge profits to commercial banks and make a high contribution to commercial banks. This is also the reason why commercial banks try to choose customers with large assets and high contribution when selecting customers. However, it also shows that commercial banks have abandoned some small- and medium-sized customers, and there is a large gap in the service of small- and medium-sized customers. When financing, from the perspective of their own development, commercial banks will take more initiative to develop large-scale enterprises with large operation scale and high-risk rating and rely on them to bring sufficient profits [22]. For small- and medium-sized enterprises, their credit rating is relatively low and their scale is small, which can not only bring limited profits to commercial banks but also face a certain risk of default. According to the research results of a platform, as shown in Figure 2, the financing needs of most enterprises are relatively low, concentrated in the range of 210000–500000 yuan; the proportion of enterprise customers with financing needs of less than 500000 yuan reached 55.3%, and the proportion of enterprises with financing needs of less than 2 million yuan reached 87.3%. It can be seen that the current financing demand of enterprises is relatively low, which deviates from the hope of commercial banks to provide large amounts of credit funds, and also creates a good development space and market demand for Internet finance.

The rapid development of information technology, especially the rapid development of Internet technology in recent years, has led to the emergence of new generation technologies such as social networks, search engines, cloud computing, and big data, which provides an effective channel to solve the problem of information asymmetry in the financial market. Through Internet channels and big data technology, the information between fund demanders and suppliers is more matched, the information asymmetry in the market is gradually transparent, and the two sides of fund supply and demand can communicate directly through the Internet platform. In addition, two or more parties can conduct transactions at the same time, making the price determination more transparent. While greatly reducing the information processing cost and transaction cost, it can form a long-term stable relationship between various participants and avoid moral hazard, as shown in Table 1. It can be seen that the fairness and effectiveness of Internet finance have been significantly improved compared with traditional finance.

Internet finance can greatly optimize the ecological environment of social capital and further improve the functional chain of investment and financing finance. According to the data of the central bank, in 2021, the proportion of China's direct financing in the scale of social financing was only 27%. The emerging direct financing channels represented by P2P online loan and equity crowdfunding have huge development space, which can innovate the investment and loan linkage in the field of Internet finance. In addition, Internet finance has seven business forms, such as P2P online loan, crowdfunding, third-party payment, and Internet fund. It has built a thinking mode of investment loan linkage and a complete financial ecosystem and has a relatively complete service closed-loop entrepreneurial service platform. Compared with traditional financial institutions such as commercial banks, Internet finance also has the advantages of fast transformation speed, strong innovation ability, low service cost, and high information transparency [23]. At the same time, Internet finance and technology finance are the most repetitive financial concepts. Internet finance can not only form a closed loop for investment and loan linkage but also integrate with traditional investment and loan institutions to jointly promote the development of technology finance and promote scientific and technological innovation.

**Figure 3:**
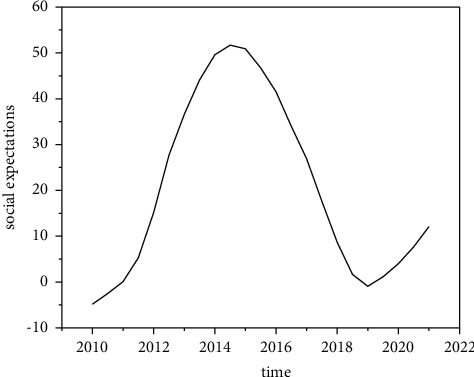
Schematic diagram of P2P development process.

**Figure 4:**
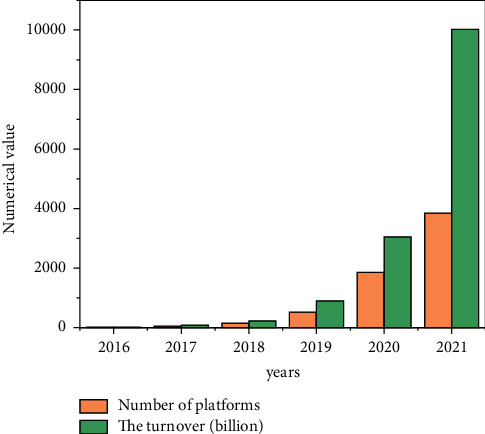
Number of P2P lending companies in China from 2016 to 2021.

**Figure 5:**
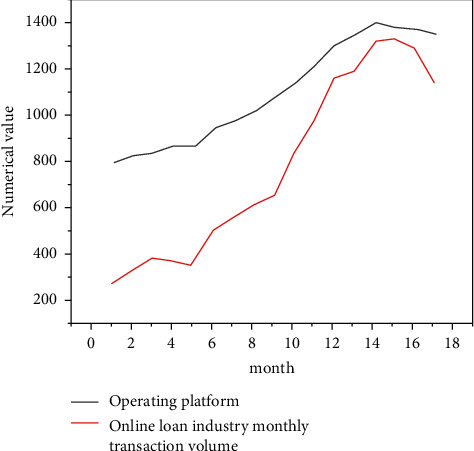
Change trend of monthly trading volume and number of operating platforms of P2P online loan industry.

**Figure 6:**
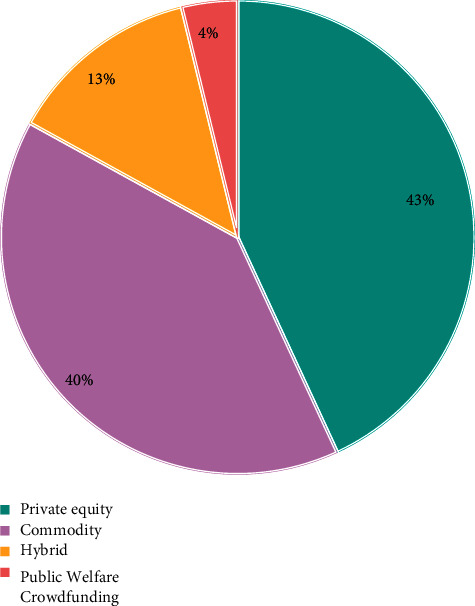
Distribution of types of crowdfunding platforms.

1. P2P Online Loan—Debt Investment. P2P loan is a relatively perfect Internet financial model. It entered China in 2007. At present, it is gradually localized in combination with China's special national conditions. According to the development process of P2P loans in China, P2P loans are currently in the period of industry integration, as shown in Figure 3.
2. In 2021, there were 6761 P2P loan companies in China, with a year-on-year increase of 138.31%; the transaction volume reached 3702.1 billion yuan, a year-on-year increase of 281.34%, showing an explosive and rapid growth trend as shown in Figure 4. Since 2016, P2P loans have been growing rapidly. The rapid development of information technology has given sufficient support. On the one hand, there is a huge demand for credit, especially for groups marginalized by banks, such as small and micro-enterprises, individual industrial and commercial households, and ordinary individuals.
3. P2P network lending mode refers to the lending relationship between individuals; that is, the investment and financing parties implement transactions based on P2P platform. Therefore, P2P network lending platform should be an information intermediary, and this investment method belongs to debt investment. As shown in Figure 5, the trading volume of P2P online loan industry is still expanding, so the online loan model can play the role of “debt investment” and play a great role in investment and loan linkage. In practice, the P2P online loan industry does invest a lot of financial resources in technology, new energy, and other enterprises, and more than 90% of the funds serve small and micro-enterprises [24]. At the same time, the P2P online loan industry still maintains a rapid development trend, and the industry scale continues to expand and has growing credit resources, which has laid a good financial foundation for the development of investment loan linkage business.
4. Equity Crowdfunding—Equity Investment. Equity crowdfunding refers to that a start-up company takes out part of its equity, and investors can obtain shares by taking shares in the company and get corresponding income in the later stage. Equity crowdfunding can play the role of “equity investment” in the linkage of investment and loan. In 2021, private equity crowdfunding platforms accounted for 41.5%, and their industry scale reached 6.7 billion∼7.3 billion, 4∼5 times that of the previous year. Moreover, the industry-leading platforms mainly serve scientific and technological small and micro-enterprises (see Figure 6). Once the investment and loan linkage service is opened, the equity crowdfunding platform can further improve the successful financing probability and efficiency of high-quality entrepreneurial projects and improve the service ability of the platform for more high-quality projects. At the same time, taking the investment and loan linkage business as an opportunity, equity crowdfunding can connect banks and other financial institutions, which helps the equity crowdfunding platform open new cooperation space between multilevel capital markets and increase business cooperation opportunities with multiple parties. In addition, with the help of investment and loan combination, the equity crowdfunding platform can also integrate the project resources, professional knowledge, and investment ability of relevant parties, which is conducive to the risk control management of the platform itself and improve the investment experience of investors.
5. Complementary Functions of Other Business Formats. In the process of capital flow, the third-party payment can complete the currency payment, capital clearing, query, and statistics between the investment and financing parties and financial institutions by providing capital circulation channels and can carry out real-time tracking analysis and early warning based on the transaction data. Internet trust and Internet fund can raise funds through the platform, invest in specific technology-based small- and medium-sized enterprises in the form of creditor's rights, and absorb government funds, venture capital institutions, social investors, etc., to guarantee the participation of companies and the participation of government credit, improve the credit rating of trust fund, and hand it over to the bank for fund custody. Internet insurance can explore the “insurance + credit” model, take credit guarantee insurance products as the carrier, give play to the financing and credit enhancement function of credit guarantee insurance, and alleviate the financing difficulties and expensive problems of small- and medium-sized enterprises. In addition, some Internet financial enterprises also have financing platforms, investment and financing advisory services (FA), media, research institutes, and other major business segments. Several business boards are connected with each other, creating a complete business closed loop for the venture capital ecosystem. They can provide products and services for entrepreneurs, investment institutions and individual investors at different stages from project collection, project incubation, project investment and financing services, project dissemination to industry research, and project packaging.

## 4. Results and Analysis

### 4.1. Reasons for the Passive Development of Commercial Banks in the Context of Internet Finance

**Figure 7:**
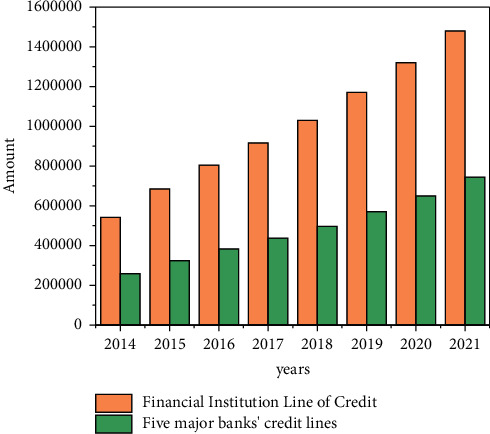
Proportion of loan scale of five major banks in 2014–2021.

The five major state-owned commercial banks *A*, *B*, *C*, *D,* and *E* have long occupied a monopoly position in the operation of China's banking industry and accounted for a large proportion of the credit amount of all financial institutions. As can be seen from Figure 7, since 2014, the credit amount of China's financial institutions has increased from 157840.5 billion yuan to 251624.1 billion yuan in 2021, with an obvious growth rate; at the same time, the credit volume of the five major state-owned commercial banks also maintained a rapid growth trend, from 61305 billion yuan in 2014 to 97312 billion yuan. Overall, the proportion of the credit volume of the five major state-owned commercial banks has maintained a downward trend, but the overall proportion is still more than 50%, indicating that their monopoly position in the domestic credit market has not changed.

**Figure 8:**
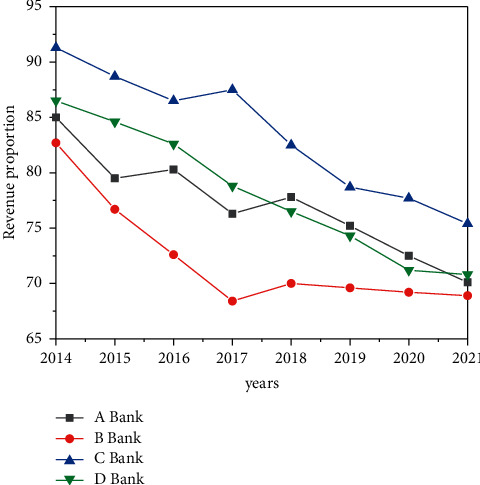
Proportion of interest margin income of commercial banks (%).

From the specific data, since 2021, the proportion of interest margin income of China's major commercial banks has gradually decreased, as shown in Figure 8. By 2021, the proportion of interest margin income of major commercial banks had basically decreased from 71% to 74%, nearly 17 percentage points lower than that in 2014. Among them, in 2021, the proportion of interest margin income of Bank of China was the lowest, reaching 51.4%, which was at a relatively leading level. It can be seen that the overall development trend is good, but the overall level is still not high, and there is still a large gap from the level of foreign commercial banks.

**Figure 9:**
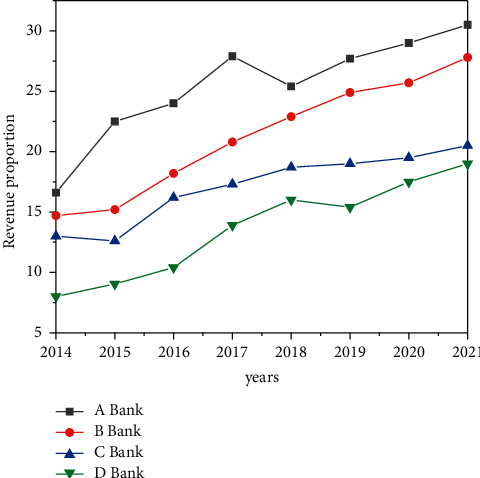
Proportion of intermediate business income of commercial banks (%).

From the perspective of intermediary business income, the income of China's major commercial banks accounted for a small proportion in 2014. For example, only 7.1% of the income of bank came from intermediary business; the highest is bank C, which only reached 16.7%. After recent development, the proportion of intermediary business income of commercial banks has significantly increased. By 2021, the proportion of intermediary business income of commercial banks is basically close to 25%, and Bank C has reached 30.6%, which is in the leading position of commercial banks, as shown in Figure 9. Compared with commercial banks in other countries, the proportion of intermediate business income of Chinese commercial banks is still relatively low, but it has maintained a good development trend. It is believed that the gap with foreign countries will be further narrowed in the future.

**Figure 10:**
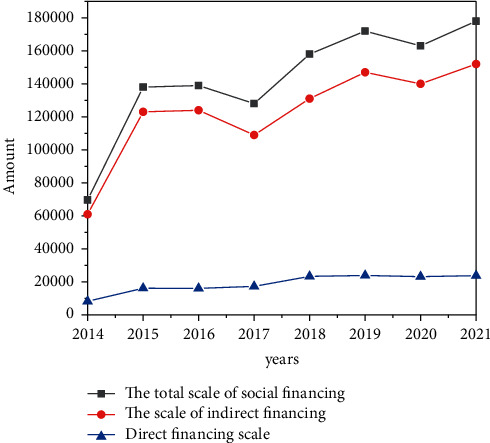
China's social financing structure from 2014 to 2021 (100 million yuan).

At present, the development level of China's financial market is still relatively low, commercial bank financing is still the main financing channel in China, and the imbalance between direct financing and indirect financing is serious, as shown in Figure 10. In China's financing structure in recent years, indirect financing has always accounted for the largest proportion, and its proportion reached the highest value of 89.4% in 2016, occupying an overwhelming advantage in the whole financing system. After that, although its proportion has declined to a certain extent, the proportion of indirect financing was 82.1% in 2021, and there is still a huge gap between the proportion of direct financing and it, which needs to be further improved.

### 4.2. Realization Mechanism of Connection between Science and Technology and Capital Market

**Figure 11:**
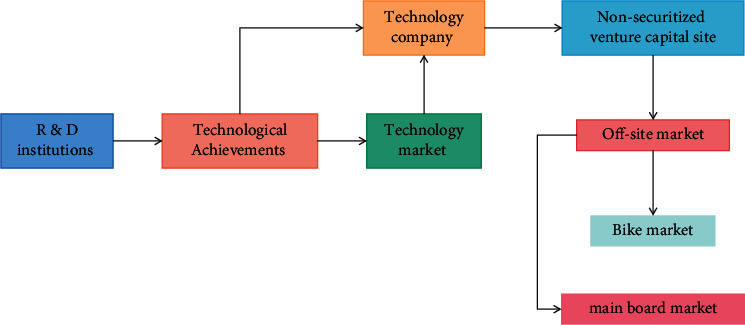
Realization mechanism of docking between science and technology and capital market.

The remarkable feature of scientific and technological achievements is their strong timeliness. The update speed of scientific and technological products is fast. If the transformation is not timely, or the application scale is too small and narrow, it may be quickly obsolete and replaced by other products or technologies. The alternative products will ruthlessly occupy the market share of the originator, and the value of scientific and technological achievements will be rapidly reduced or even lost. It can be seen that the transformation of scientific and technological achievements is not only a question of whether they can be commercialized but also a question of whether they can quickly occupy the market, realize large-scale application, and maximize their value in a short time. This inevitably requires strong capital support, and only the capital market can provide such support. Therefore, the transformation of scientific and technological achievements and the industrialization of scientific and technological achievements must connect with the capital market and must rely on and make use of the capital market. This is the only way for the transformation and industrialization of scientific and technological achievements. The realization process and mechanism of the connection between science and technology and capital market are shown in Figure 11.

In the early stage of the industrialization of scientific and technological achievements, absorbing venture capital is an important aspect of the industrialization of scientific and technological achievements. In particular, the absorption of organized venture capital can not only bring long-term capital but also bring value-added services such as management experience, financing, and sales channels.

**Figure 12:**
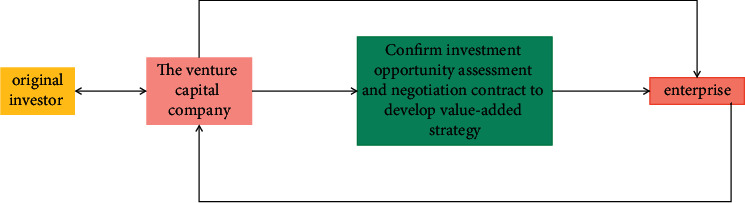
Investment mechanism of venture capital institutions.

Organized venture capital is invested through venture capital institutions. The process usually includes options, negotiation, contract determination, and post-investment management, as shown in Figure 12. Its investment system design also focuses on these aspects.

As the main body and carrier of scientific and technological innovation activities, science and technology enterprises can use the property right trading market to transfer part of their equity, technology patents (intangible assets), and tangible assets in the property right trading market and can also list and finance science and technology projects or patents, which provides a new financing channel to solve the capital shortage of small- and medium-sized science and technology enterprises and lays a good foundation for further capital market operation. The transfer of equity, patents, and tangible assets operates basically the same in the property rights trading market.

#### 4.2.1. Equity Transfer of Science and Technology Enterprises

**Figure 13:**
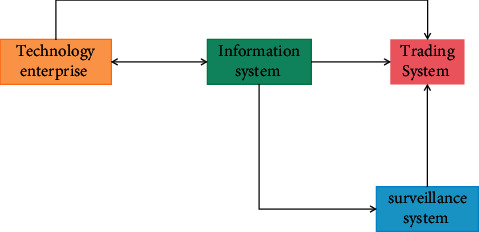
Operation mechanism of equity transfer of science and technology enterprises in property right trading market.

Under the condition of non-listing, through the transfer of equity in the property right trading market, science and technology enterprises meet the purposes of investment withdrawal, improving equity structure and introducing strategic investors in the transformation of scientific and technological achievements, which is conducive to accelerating the transformation of scientific and technological achievements. The basic operation process is shown in Figure 13.

The first step is that the technology enterprises should trust the equity of the enterprise in the designated custodian institution, sign an agreement with the property right brokerage institution and entrust it to conduct property right transactions; the second step is to entrust the appraisal institution to evaluate its equity value and clarify the transfer purpose and the number of shares transferred; the third step is to promote in the relevant information system through the qualification examination and screening of the property right exchange; the fourth step is to offer the transfer price, put forward the basic and necessary conditions for the transfer, and list them in the relevant trading system; the fifth step is to preliminarily determine the collection of qualified transferees, and the final transferee is determined by bidding; and the sixth step is to sign a contract, transfer the ownership in the equity custody institution, and settle in a special account.

In this process, there are two problems that have a certain impact on the smooth realization of property right transaction. First, who is the subject of the qualification of the transferee? As a market platform, the main responsibility of the exchange is to maintain fairness and justice. However, it is not conducive to the exercise of the transferor's rights for the exchange to confirm the qualification of the transferee as a single subject, while the transferor as a single subject to confirm the qualification of the transferee may deliberately exclude other transferee intents and hinder fair trading because there are potential transferee before the transferor is listed. Therefore, the relevant mechanisms in this regard need to be studied. The exchange and the transferor jointly determine that negotiation is a way to solve this problem [25]. It also requires that when setting the necessary transfer conditions, there should be reasons based on the purpose of development and legal compliance. The proposed transfer conditions should be necessary and reasonable, and there should be no content with clear direction or against fair competition.

The second is the selection of bidding methods. In theory, online electronic bidding is the most ideal bidding mechanism. It is not limited by region and number of people and can conduct continuous bidding, but it can play the best effect only when there are more participants. As the property right transaction cannot be broken down, there will not be many bidding participants, which is difficult to ensure the full disclosure of the equity value. In fact, the number of bidding participants has become the key to the bidding mechanism. Therefore, market managers should actively publicize to introduce multiple bidders. In addition, whether we can learn from the principle of futures, establish a futures market with property price as the target, and guide the delivery price through the price of futures market is also a problem of research value.

### 4.3. Demonstration of the Relationship between Science and Technology Finance, Technological Innovation, and Economic Development

At present, the factor market with significant changes and the fierce competition among enterprises with different technologies and absolute or relative factor cost differences from different regions and countries have become the prominent characteristics of global economic operation. Among them, the key force that can cause factor changes and win the competition is technological change. There are two different but transformable dimensions of technological change, namely new general technological change and special technological change. The former can be widely used by factor users in the factor market to improve total factor productivity; the latter has a limited range of applications, but it is a new source of general technology. The introduction and adoption of technological change will bring changes in resource endowment and affect the structure of relative price, so as to realize the adjustment of industrial structure and even economic structure, so as to obtain industrial growth and economic development. Therefore, how to effectively guide the transformation of special technology, improve the transformation efficiency from special technology to general technology, and promote the application of new general technology has become the key link of economic development. As the deep integration of science and technology and finance, science and technology finance can obviously shoulder this important task. The reasons are as follows.

**Figure 14:**
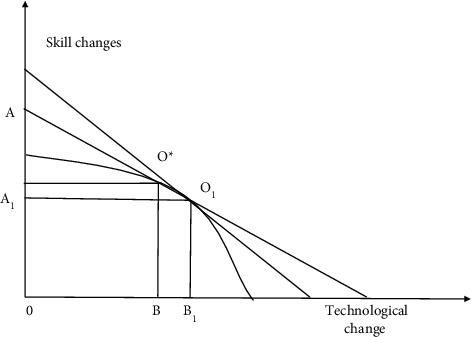
Technological change.

**Figure 15:**
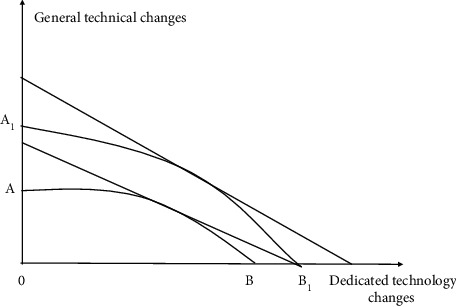
Special technology change.

Figure 14 shows two choices faced by enterprises: one is the growth brought by adjustment in the skill space defined by the existing technology, but the conversion cost of skill change needs to be considered; the second is the growth brought by the introduction of new technological changes to reconstruct the skill space, but there will also be greater change costs. Therefore, the boundary of possible change is formed by the effects of investing in skill change and technological change.  Figure 15 compares the types of technological change, i.e., special technology and general technology, to illustrate the final benefits of these two types of technological change. Line *AO*^*∗*^*B* in Figure 14 represents the equal income line without financial support, and the intercept of the vertical axis and horizontal axis represents the level of skill and technological change, respectively. Line *A*_1_*O*_1_*B*_1_ represents the change after financial support. The adjustment cost of technological change is reduced through financial support, which benefits from the improvement of capital availability and the expansion of total capital. Therefore, enterprises are more willing to choose technological change that changes the original skill space, which will lead to the expansion of output boundary. In Figure 15, through the credit screening of financial institutions and the rational selection of financial markets, it can not only greatly improve the identification efficiency of which special technology can be more effectively converted into general technology but also improve the promotion efficiency of the introduction and application of general technology, to improve the quantity, variety, and influence of technological innovation [26]. It is realized as the expansion of the output boundary from *AB* to *A*_1_*B*_1_ and the growth of the overall level of the equal income line.

## 5. Conclusion

The combination of science and technology and finance refers to the mutual support and interaction between science and technology activities and financial activities. This study mainly studies the support and promotion of the financial system to science and technology. To study the mechanism of the combination of science and technology and finance is to study the structure, relationship, and connection of relevant subjects in the process of the combination of science and technology and finance, as well as the way of interaction, and to study the protective and promoting role of financial instruments, financial institutions and environment in the generation, and transformation of scientific and technological achievements. In modern economy, science and technology and finance have become the most active factors in today's social productive forces. The development of science and technology is inseparable from the support of finance. On the one hand, to win the initiative in the competition, relying on scientific and technological innovation to improve the country's comprehensive national strength and core competitiveness, establish a national innovation system, and take the road of innovative national development has become the common choice of many countries in the world. On the other hand, the modern financial industry has developed into a deeper and broader field. Financial institutions have provided new support and vitality for the development of science and technology through financial innovation methods such as redesign, improvement, and recombination, which have greatly promoted the development of modern high technology. Commercial banks have both advantages and disadvantages in the process of competing with Internet finance. In terms of advantages, commercial banks have more customer resources, more risk aversion tools, and more stable operation system, and mobile payment products have achieved initial results; in terms of disadvantages, commercial banks have industry monopoly and lack of crisis awareness, which are limited to the negative response of the established profit model, insufficient product innovation and development, unreasonable capital utilization structure, and the constraints and restrictions of the macro-environment. Under the impact of Internet finance, commercial banks should take positive countermeasures, mainly including fully promoting product and service innovation, actively exploring the field of Internet financial business, building a one-stop financial service platform, fully promoting Internet technology innovation, respecting the spirit of the Internet, seeking win-win cooperation, and improving the efficiency of the internal mechanism of commercial banks.

## Figures and Tables

**Table 1:** Comparative analysis of resource allocation between traditional finance and Internet finance.

Comparison item	Traditional finance	Online finance
Information processing	High cost	Low cost
Risk assessment	Information asymmetry	Information symmetry
Capital supply and demand	Bank intermediary	The supplier and the demander shall solvethe problem by themselves
Means of payment	Banks act as payment intermediaries	Payment system
Supply and demand sides	Indirect transaction	Direct transaction
Transaction cost	Higher	Internet-based, low cost

## Data Availability

The labeled data set used to support the findings of this study is available from the author upon request.
